# Evaluation of Corrosion Behavior by Measuring Passivation Current Density of Dental Implant Coated with Bioceramic Materials

**DOI:** 10.1155/2021/9934073

**Published:** 2021-06-10

**Authors:** Hanan Ali Hameed, Haider Ali Hasan, Mohammad Khursheed Alam

**Affiliations:** ^1^Prosthodontic Department, College of Dentistry, University of Babylon, Iraq; ^2^Oral and Maxillofacial Surgery Department, College of Dentistry, University of Babylon, Iraq; ^3^Preventive Dentistry Department, College of Dentistry, Jouf University, Sakaka, Saudi Arabia

## Abstract

**Background:**

This paper reports the corrosion behavior of uncoated commercially pure titanium and Ti-6Al-4V samples and these coated with hydroxyapatite, partial stabilized zirconia (PSZ), and the mixture of partial stabilized zirconia and hydroxyl-apatite by measuring passivation current density and see if there are any differences between them using electrochemical polarization tests in 37°C Hank's solution.

**Materials and Methods:**

The electrophoretic deposition method (EPD) was elected to keep the coating materials which are HA, PSZ, and the mixture of 50% HA and 50% PSZ on Cp Ti and Ti-6Al-4V alloy samples. The electrochemical corrosion test was achieved by exposing the coated and uncoated samples to Hank's solution which prepared in the laboratory and measuring the polarization potential, passivation current density, and the open circuit potential for all samples.

**Results:**

The results indicated that the passivation current density for all Cp Ti and Ti-6Al-4V alloy groups that coated with HA, PSZ, and with mixture of 50/50 HA and PSZ was less than uncoated groups. There are no significant differences between all Cp Ti groups when compared with all Ti-6Al-4 V alloy groups. The open circuit potential (OCP) for both Cp Ti and Ti -6Al -4V samples was in the following sequence PSZ > HA > mixture of HA and PSZ > uncoated.

**Conclusions:**

Coating significantly decreased the passivation current density of Cp Ti and Ti-6Al-4V alloy.

## 1. Introduction

Oral implantology offers a safe and reliable solution for replacing a missing tooth [1]. Many factors can be affecting the clinical success of implant osseointegration such as biocompatibility, mechanical properties, and corrosion resistant of implant materials. The meaning of corrosion is the action or effect of corroding products and the release of elemental constituents to the surrounding environment. There are different types of corrosion wet corrosion and dry corrosion. The dry corrosion or called chemical corrosion means direct contact nonmetallic and metallic element to form chemical compound by process of sulfurization reactions or oxidation or halogenation. The wet corrosion or called electrochemical corrosion which should present water or other fluid (electrolyte) [2, 3]. In the oral environment, the most common type is electrochemical corrosion because saliva which contain salt and act as weak electrolyte. The magnitude of electrochemical corrosion depends on many factors which influence strength of any electrolyte such as concentrations of saliva components, pH, buffering capacity, and surface tension [2].

Corrosion resistance is considered as one of the most important dental material properties and critically for implant materials. The strength and the fatigue resistance of metal alloys greatly compromised by corrosion, and this will lead to structural and mechanical failure of dental implants and their prosthetic components [4, 5]. Also, from the effects of corrosion, releasing of different sizes and characteristics of titanium particles from the dental implant surface which leads to adjacent soft tissue discoloration and local pain or swelling in the absence of infection [6].

The releasing of corrosion particles leads to loss of bone and osteolysis that in fact phagocyted by macrophages which stimulate inflammatory mediators releasing such as cytokines. The liberation of these mediators towards the bone surface initiates its resorption by activation the osteoblast [7]. Commercially pure titanium and titanium six aluminum four vanadium (Ti-6Al-4V) alloys are commonly used as implant materials and especially as dental implant material, and bioactive materials like HA were to improve implant osseointegration. It was found better bonding between the bone and implant material Ti-6Al-7Nb coated with HA or partial stabilized zirconia or mixture of both than uncoated samples when implanted in rabbit tibia [8]. This work is planned to evaluate the corrosion behavior of uncoated Cp Ti and Ti-6Al-4V implant material through evaluation the passivation current density and compared with those of coated samples with HA, PSZ and mixture of 50/50 HA and PSZ.

## 2. Materials and Methods

### 2.1. Samples Preparation

Commercially pure titanium and Ti-6Al-4 V alloy were used in this study. Commercially pure Titanium was cut into small rectangular (5 mm × 17 mm × 17 mm), and Ti-6Al-4 V alloy was cut into small rectangular samples (2 mm × 17 mm × 27 mm). Then, all samples were polished with silicon carbide paper and ultrasonic cleaned. After that, the samples were divided into four subgroups for each alloy. One of the subgroups was kept uncoated, and the others were coated with HA, PSZ, and with mixture of 50/50 HA and PSZ using the electrophoretic technique.

### 2.2. Electrophoretic Deposition

According to the type of coating materials, three suspensions were prepared. The first suspension was formulated by addition the powder of HA to the ethanol (100 g/I liter) as a solvent in a container over a stirrer without any dispersant or binder agents. Continue with stirring until a colloidal suspension was gotten [9]. The second suspension was formulated in a container over a stirrer by adding powder of PSZ to ethyl alcohol (200 g/1 liter) as a solvent. The dispersant agent which was phosphate ester (3 g/1 liter) and the binder agent which was a polyvinylbutyral (3.5 g/1 liter) were added [10]. In a container over a stirrer, the third suspension was formulated by adding powder of 50 : 50 ratio HA/PSZ to the ethyl alcohol as a solvent; after 10 minutes, the dispersant agent which was phosphate ester 3 g/1 liter was added and after stirring, the binder which was polyvinylbutyral was added (3.5 g/1) [8].

### 2.3. X-Ray Phase Analysis

The phase analysis was evaluated for the samples of Cp Ti and Ti-6Al-4 V alloy before and after using different materials of coating employing 3121 powder X-ray Diffractometer using Cu K*α* radiation. In step of one degree, the 2*θ* angles were swept from 20 to 80°.

### 2.4. Electrochemical Corrosion Test

#### 2.4.1. Preparation of Electrolyte Solution

High purity reagents which are NaCl, KCl, CaCl, MgSo_4_.7H_2_O, NaH_2_PO_4_.2H_2_O, NaHCO_3_, glucase, KH_2_PO_4_, and MgCl_2_.6H_2_O were used to prepare Hank's solution. The solution was maintained at 37 ± 1°C by using a water path [11].

### 2.5. Tafel Extrapolation

Electrochemical testing was performed in a potentiostat and a standard glass cell containing three electrodes; the first electrode was platinum counter electrode CE, the second electrode was a working electrode WE (specimen), and the third electrode was a reference electrode RE. The specimen was fixed on orifice with diameter (1 cm) on the side of the glass cell, and the specimen was exposed to solution throw this orifice only for one hour.

**Figure 1:**
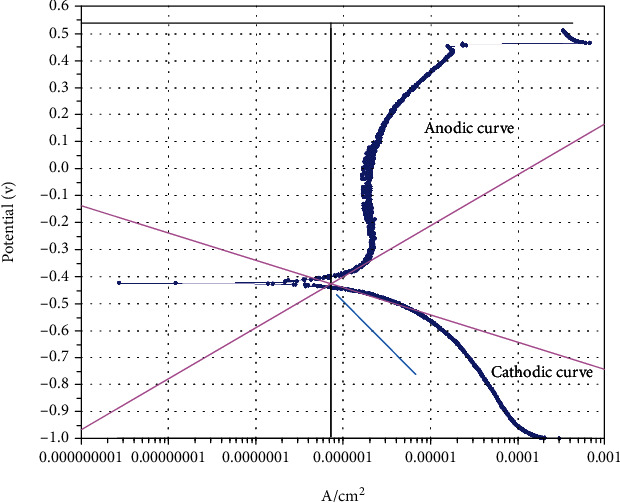
Determination passivation current density.

The passivation current density was determined from the graph at 0.5 V potential and by drawing horizontal line at 0.5 V intercepting the anodic curve. From the point of interception, a vertical line was drawn to intercept the horizontal axis. This point is considered the passivation current density as shown in Figure 1.

## 3. Results

### 3.1. X-Ray Diffraction of Coated Samples

The XRD patterns of Ti-6Al-4 V samples were coated with HA uncoated samples. At 2*θ*, strong line of *α* Ti showed with the pattern of uncoated Ti-6Al-4 V samples. For the HA-coated samples, strong line of HA was showed in the XRD results. The XRD patterns of Ti-6Al-4 V alloy coated with PSZ showed that surface of samples was well covered with PSZ when compared with the uncoated sample. The domination of PSZ in the coated layers showed for the samples coated with mixture of HA powder and PSZ.

Strong line of *α* Ti showed with the pattern of uncoated Cp Ti samples. For the HA coated samples, strong line of HA was showed in the XRD results. The XRD patterns of Cp Ti alloy coated with PSZ showed that surface of samples was well covered with PSZ when compared with uncoated sample. The domination of PSZ in the coated layers showed for the samples coated with mixture of HA powder and PSZ.

### 3.2. Corrosion Test

#### 3.2.1. Open Circuit Potential (OCP)

**Figure 2:**
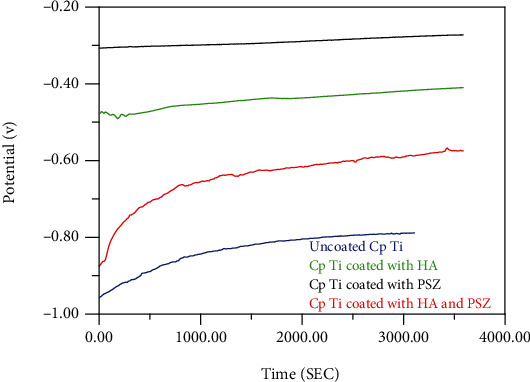
Open circuit potential for uncoated and coated Cp Ti with HA, PSZ, and mixture of HA and PSZ.

**Figure 3:**
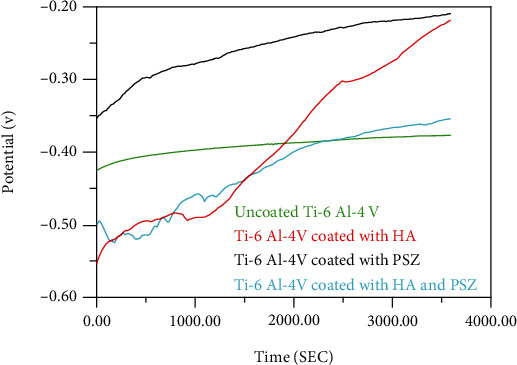
Open circuit potential for uncoated and coated Ti-6Al-4 V alloy with HA, PSZ, and mixture of HA and PSZ.

The open circuit potential for coated samples using several types of materials and uncoated samples was shown in Figures 2 and 3. The lower the OCP, the less resistance to corrosion, and therefore, the samples were in the following sequence from less corrosion resistance to the highest: uncoated (Cp Ti = −0.383 V, Ti − 6Al − 4V = −0.825 V) < mixture (Cp Ti = −0.358 V, Ti − 6Al − 4V = −0.550 V) < HA (Cp Ti = −0.225 V, Ti − 6Al − 4V = −0.40 V) < PSZ (Cp Ti = −0.212 V, Ti − 6Al − 4V = −0.265 V).

### 3.3. Passivation Current Density of CP Ti and Ti-6Al-4V Alloy

Different coatings were applied (HA, PSZ, and mixture of HA and PSZ) on both alloys, and comparison was done between them. The mean and variance for all groups are listed in Table 1.

**Figure 4:**
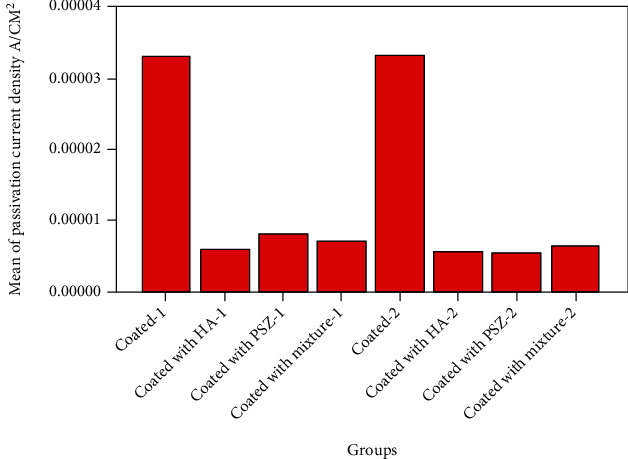
Bar chart plot for mean values of the passivation current density of Ti-6Al-4 V (1) alloy and Cp Ti (2) with different coatings.

Figure 4 illustrates the bar chart for mean values of the passivation current density parameter for coated and uncoated samples for the two of the implant's materials. Analysis of variances and means test showed highly significant difference among groups of Cp Ti and Ti-6Al-4 V alloy samples as shown in Table 2.

The results indicating that there are highly significant differences at *P* < 0.01 have been pointed among different variances as well as for different means and that should conclude at least one pair of groups that are not equal and that need to be continuing the comparisons by using the least significant difference (LSD) method, as illustrated in Table 3.

## 4. Discussion

### 4.1. Passivation Current Density of Cp Ti and Ti-6Al-4V Alloys

There is no significant differences between uncoated Ti-6Al-4V alloy and uncoated Cp Ti this may be due to both alloys formed passive film which act as insulting barrier. There are highly significant differences between passivation current density of uncoated Ti-6Al-4 V alloy and Cp Ti coated with HA and PSZ and mixture of HA & PSZ this is may be due to corrosion resistance of Ti-6Al-4 V alloy is less than Cp Ti and furthermore Cp Ti is protected with HA, PSZ and mixture of HA & PSZ which is thin layer of coating materials changing the surface chemistry due to precipitation of coating compound and have high corrosion resistance property. The results of the study show highly significant differences between Ti-6Al-4 V alloy coated with (HA and PSZ and mixture of HA & PSZ) and uncoated Cp Ti this is may be due to treated and protected the alloy by previous coating materials which are high shifts to noble values, this suggests the formation of a passive film. The formed film acts as a barrier for metal dissolution and thus, can reduce the corrosion rate. There are no significant differences between all coated groups of Cp Ti when compared with all coated Ti-6Al-4 V alloy, this may be due to powerful insulting effect of all coating materials which act as barrier between the substrate surface (Cp Ti, Ti-6A-4 V) and the solution of the body fluid. Also, the effective bond between all coating and the surface of substrate.

### 4.2. Passivation Current Density of Cp Ti

There is highly significant differences between passivation current density of uncoated Cp Ti and Cp Ti coated with HA, and this is may be due to protect the samples by HA and that make it more resistance to corrosion by formation passive film at early stages, and this agreed with [12] stated highly significant differences between passivation current density uncoated Cp Ti (I passive 4.6 × 10^−5^ A/cm^2^ in 4.5 V) and Cp Ti coated with HA (I passive 1.4 × 10^−6^ A/cm^2^ in 4.5 V) in Hank's solution at 37°C. And this disagreed with [13] stated that the cyclic polarization curves were obtained for uncoated and HA coated CP–Ti. The passivation current density of the uncoated sample was marginally lower than that of the coated samples. At 1.5 V, within the passive region, current densities of 3.47 × 10^−6^ A/cm^2^ and 8.02 × 10^−6^ A/cm^2^ were recorded for the uncoated and coated samples, respectively. The smaller size of the loops obtained for coated samples indicates the stability of the HA/Ti interface against corrosion attack and the faster repassivation tendency of the HA coatings; the disagreement may be due to the electrochemical corrosion test done in the Ringer solution while the SBF was used in this study. And the same conclusion stated by [3] that he found that the smallest value of passive current density was shown by uncoated Cp Ti (about 0.1 × 10^−4^ A/cm^2^) in artificial saliva solution at 25°C. The disagreement may be due to the electrochemical corrosion test done in artificial saliva solution at 25°C, and the biomimetic method was used to coat the cp Ti surface with hydroxyapatite. The result of the study shows that there are highly significant differences between uncoated Cp Ti and Cp Ti coated with PSZ and mixture of HA and PSZ, and this is may be due to that coating materials reached to the passive state at early time than uncoated which indicates good corrosion resistance. There are no significant differences between all coated groups of Cp Ti, and this may be due to the coating layer which formed an insulting layer to protect the metal from delivering electrons.

### 4.3. Passivation Current Density of Ti-6Al-4V Alloy

There is a highly significant difference between passivation current density of uncoated Ti-6Al-4V alloy and Ti-6Al-4V alloy coated with HA, and this agreed with [14] stated that there is a highly significant difference between passivation current density that was exhibited by the HA coated Ti-6Al-4V samples by sol-gel in Ringer's solution at room temperature more than uncoated samples. The passivation current density was for uncoated Ti-6Al-4 V alloy (9.5 × 10^−4^ A/cm^2^) and for Ti-6Al-4 V coated with HA (1.5 × 10^−4^ A/cm^2^). There is a highly significant difference between passivation current density of uncoated Ti-6Al-4V alloy and Ti-6Al-4 V alloy coated PSZ and mixture of HA and PSZ, and this is may be due to that coating materials reached to the passive state at early time than uncoated which indicate good corrosion resistance. There are no significant differences between all coated groups of Cp Ti, and this may be due to the coating layer which formed an insulting layer to protect the metal from delivering electrons.

## Figures and Tables

**Table 1:** Summary statistics (means and variances) of passivation current density parameter for coated and uncoated Ti-6Al-4V alloy and CP Ti in *μ*A/cm^2^.

Groups	No.	Ti-6A1-4V alloy-1		Cp Ti-2	
		Mean × 10^−5^	S.D.×10^−5^	Mean × 10^−5^	S.D.×10^−6^
Uncoated	8	3.320	1.520	3.330	6.14
Coating with HA	8	0.607	0.141	0.575	4.25
Coating with PSZ	8	0.816	0.277	0.551	1.27
Mixture of PSZ and HA	8	0.727	0.334	0.655	2.36

**Table 2:** Coincidence's tests for parameters (variances and mean) between different treated materials according to the “Passivation current density “parameter.

Criteria	Test of homogeneity of variances (*σ*^2^)		ANOVA test of equality of means (*μ*)	
	Levene statistic	Sig.		Sig.^(^^∗^^)^
Passivation current density	8.925	0.000	31.069	0.000

**Table 3:** Multiple comparison by (LSD) among all pairs for passivation current density parameter in the two materials according to different treated groups.

(I) groups	(J) groups	Sig.^(^^∗^^)^	C.S.
Uncoated-1	Coating with HA-1	0.000	HS
	Coating with zirconia-1	0.000	HS
	Mixture of zirconia and HA-1	0.000	HS
	Uncoated-2	0.963	NS
	Coating with HA-2	0.000	HS
	Coating with zirconia-2	0.000	HS
	Mixture of zirconia and HA-2	0.000	HS
Coating with HA-1	Coating with zirconia-1	0.508	NS
	Mixture of zirconia and HA-1	0.703	NS
	Uncoated-2	0.000	HS
	Coating with HA-2	0.920	NS
	Coating with zirconia-2	0.860	NS
	Mixture of zirconia and HA-2	0.879	NS
Coating with zirconia-1	Mixture of zirconia and HA-1	0.778	NS
	Uncoated-2	0.000	HS
	Coating with HA-2	0.446	NS
	Coating with zirconia-2	0.402	NS
	Mixture of zirconia and HA-2	0.610	NS
Mixture of zirconia and HA-1	Uncoated-2	0.000	HS
	Coating with HA-2	0.630	NS
	Coating with zirconia-2	0.577	NS
	Mixture of zirconia and HA-2	0.819	NS
Uncoated-2	Coating with HA-2	0.000	HS
	Coating with zirconia-2	0.000	HS
	Mixture of zirconia and HA-2	0.000	HS
Coating with HA-2	Coating with zirconia-2	0.000	NS
	Mixture of zirconia and HA-2	0.939	NS
Coating with zirconia-2	Mixture of zirconia and HA-2	0.801	NS

^(^^∗^^)^HS: highly significant at *P* < 0.01; NS: nonsignificant at *P* > 0.05.

## Data Availability

All data are available within the manuscript. Raw data can be made available upon reasonable request.
